# Sociodemographic profile, diagnoses and nursing care in post-COVID-19 patients in a Brazilian university hospital

**DOI:** 10.1590/0034-7167-2022-0730

**Published:** 2023-07-31

**Authors:** Evelyn Klein dos Santos, Fernando Riegel, Jhonatan Tyson Barros Azevedo, Maria da Graça Oliveira Crossetti, Margarita Ana Rubin Unicovsky, Jussara Gue Martini, Paula Bresolin, Andrea Aparecida Gonçalves Nes

**Affiliations:** I Universidade Federal do Rio Grande do Sul. Porto Alegre, Rio Grande do Sul, Brazil; II Universidade Federal de Santa Catarina. Florianópolis, Santa Catarina, Brazil; III Lovisenberg Diaconal University College. Oslo, Norway

**Keywords:** Pandemic, Nursing Diagnosis, Nursing Care, COVID-19, Nursing, Pandemia, Diagnósticos de Enfermería, Cuidados de Enfermería, COVID-19, Enfermería, Pandemia, Diagnósticos de Enfermagem, Cuidados de Enfermagem, COVID-19, Enfermagem

## Abstract

**Objectives:**

to analyze the sociodemographic profile, diagnoses and nursing care of post-COVID-19 patients admitted to a university hospital in southern Brazil.

**Methods:**

a retrospective cohort study. The sample consisted of 1,467 medical records, from January 2020 to January 2021.

**Results:**

from the analyzed medical records, the most prevalent profiles, respectively, included: males (52.9%); white (81.1%); with Impaired Physiological Balance Syndrome* Nursing Diagnosis (77.3%); nursing care implementing aerosol precautions (94.5%); implementing droplet precautions (93.4%); checking vital signs (91.9%); applying standard disinfectant to equipment and surfaces (89.6%).

**Conclusions:**

the analysis of the sociodemographic profile, diagnoses and nursing care of patients in this study may contribute to implementing the Nursing Process in the coronavirus pandemic context.

## RESUMO

**Objetivos:**

analisar o perfil sociodemográfico, diagnósticos e cuidados de enfermagem de pacientes pós-COVID-19 internados em um hospital universitário do sul do Brasil.

**Métodos:**

estudo de coorte retrospectivo. A amostra foi constituída de 1.467 prontuários, no período de janeiro de 2020 a janeiro de 2021.

**Resultados:**

dos prontuários analisados, os perfis, respectivamente, mais prevalentes incluíram: pessoas do sexo masculino (52,9%); da cor branca (81,1%); com Diagnóstico de Enfermagem de síndrome do equilíbrio fisiológico prejudicado* (77,3%); cuidados de enfermagem implementar medidas de precauções por aerossóis (94,5%); implementar medidas de precaução por gotículas (93,4%); verificar sinais vitais (91,9%); aplicar desinfetante padrão em equipamentos e superfícies (89,6%).

**Conclusões:**

a análise do perfil sociodemográfico, diagnósticos e cuidados de enfermagem dos pacientes deste estudo poderá contribuir à implementação do Processo de Enfermagem no contexto pandêmico do coronavírus.

## RESUMEN

**Objetivos:**

analizar el perfil sociodemográfico, diagnósticos y cuidados de enfermería de pacientes post-COVID-19 internados en un hospital universitario del sur de Brasil.

**Métodos:**

un estudio de cohorte retrospectivo. La muestra estuvo conformada por 1.467 historias clínicas, desde enero de 2020 hasta enero de 2021.

**Resultados:**

de las historias clínicas analizadas, los perfiles más prevalentes, respectivamente, incluyeron: hombres (52,9%); blanco (81,1%); con diagnóstico de enfermería de síndrome del equilibrio fisiológico alterado* (77,3%); los cuidados de enfermería implementan medidas de precaución por aerosoles (94,5%); implementar medidas de precaución por gotitas (93,4%); control de signos vitales (91,9%); aplicar desinfectante estándar a equipos y superficies (89,6%).

**Conclusiones:**

el análisis del perfil sociodemográfico, diagnósticos y cuidados de enfermería de los pacientes de este estudio puede contribuir para la implementación del Proceso de Enfermería en el contexto de pandemia del coronavirus.

## INTRODUCTION

According to the World Health Organization (WHO), COVID-19 can be defined as an infectious disease caused by the SARS-COV-2 coronavirus, identified in December 2019 in Wuhan, China. Due to its rapid spread across continents, on March 11, 2020, the WHO defined the situation as a COVID-19 pandemic. By July 2022, approximately 574 million cases and 6.39 million deaths were confirmed worldwide^(1)^.

Most cases of COVID-19 are asymptomatic or mild, with the main signs and symptoms being cough, fever, body pain, fatigue, hemoptysis, dyspnea and diarrhea. In more severe cases, patients may experience pneumonia, acute respiratory distress syndrome (ARDS), acute heart problems, multiple organ failure and death. However, as the pandemic affects more people, reports related to neurological symptoms have increased. Among these reports, headache, anosmia, dysgeusia, dizziness, seizures and impaired consciousness stand out^(2)^.

Infection with the disease in question can cause complications in the host organism, such as encephalitis, systemic inflammation, peripheral organ dysfunction and cerebrovascular changes, which can lead to long-term neurological sequel, which can aggravate pre-existing symptoms or initiate new conditions and cerebro-vascular diseases such as stroke. Moreover, research indicates that one third of survivors have cognitive or motor impairment at the time of discharge, being more pronounced in risk groups^(3)^.

In the meantime, the nurse role in the application of the Nursing Process (NP) is highlighted with the purpose of identifying and meeting the care needs of patients affected by COVID-19, mitigating complications and sequel caused by the infection^(4)^. In this regard, nurses assist their patients holistically, aiming to adapt nursing care prescription to the specificities that the coronavirus infection imposes.

The NP can be defined as a methodological instrument that guides care and is structured in five stages, namely: nursing history; Nursing Diagnosis (ND); nursing planning; nursing implementation; and nursing assessment^(5)^. In this regard, it is important to highlight the importance of applying the NP in daily nursing care, such as in times of a pandemic, contributing to the high professional care quality and safety in adverse and complex situations.

The justification for carrying out the study is based on the lack of research involving NP implementation in post-COVID-19 patients, with a view to improving the quality of care provided. In this direction, this study is relevant, considering that in the nursing team’s daily work the elaboration of care prescriptions focused on the needs and uniqueness of patients surviving COVID-19 is required, aiming at mitigating the deficits caused by the disease^(3, 4)^. In view of this, the guiding question of this study stands out: what is the sociodemographic profile, diagnoses and nursing care of post-COVID-19 patients admitted to a university hospital in southern Brazil, from January/2020 to January/2021?

## OBJECTIVES

To analyze the sociodemographic profile, diagnosis and nursing care of post-COVID-19 patients admitted to a university hospital in southern Brazil.

## METHODS

### Ethical aspects

The research project was submitted to the Nursing School Research Commission (COMPESQ) and the Research Ethics Committee (REC) of the *Hospital de Clínicas de Porto Alegre* (HCPA), which has regulatory guidelines for research involving human beings and guidelines for procedures in research with any stage in a virtual environment^(6, 7, 8)^. As this is a study that used a secondary database, the Informed Consent Form was waived.

### Study design, period and place

This is a retrospec tive cohor t study^(9)^ that followed the STrengthening the Reporting of OBservational studies in Epidemiology (STROBE) recommendations. The field of research was HCPA Post-COVID-19 Clinical Inpatient Units. *Hospital de Clínicas* is a public university hospital linked to the Ministry of Education and the *Universidade Federal do Rio Grande do Sul* (UFRGS), with 919 beds and 6,843 employees. During the COVID-19 pandemic, it was one of the reference hospitals for caring for critically ill patients with COVID-19. The hospital has a 62-bed Adult Intensive Care and Coronary Care Unit and 135 beds for the COVID-19 adult Critical Care Units (CCU)^(10)^.

### Data collection, sample, inclusion and exclusion criteria

The research sample consisted of 1467 medical records of COVID-19 survivors who were hospitalized in HCPA Clinical Units from January 2020 to January 2021.

Data were requested through a query of the clinical database associated with HCPA’s COVID-19 Biobank^(11)^ between April and July 2022. Inclusion criteria were COVID-19 survivors, aged 18 years or older, of both sexes, after hospitalization in a CCU. Exclusion criteria were surviving COVID-19 patients under the age of 18 who were not admitted to the CCU and medical records with incomplete information.

### Analysis of results

Qualitative variables were analyzed and presented in terms of absolute and relative frequencies [n (%)] and quantitative variables in terms of mean, standard deviation, (quartile 1 – quartile 3), lowest and highest values and missing data count. Quantitative variable distribution was assessed through histogram graphical analysis and the quantile-quantile graph. Due to the high asymmetry in the distribution of the studied variables, the difference between groups was performed using the Kruskall-Wallis test. All analyzes were performed in R software, version 4.2.0, using the tidyverse package, version 1.3.1^(12, 13)^. Figures were created in Scalable Vector Graphics format.

## RESULTS

The research analyzed 1467 medical records of patients who tested positive for SARS-CoV-2 after discharge from the CCU from January 2020 to January 2021.

As for sociodemographic characteristics, there was a prevalence of males (52.9%), average age group of 58.9 years, white race/color (81.1%), married (42.9%) and retired (11.2%). Table 1 shows the sociodemographic variables of post-CUC COVID-19 survivors are presented. Regarding clinical variables, it should be noted that the most prevalent diagnostic test for COVID-19 was PCRVR, research for respiratory virus PCR, performed in (44%) 646 patients. With regard to patients’ weight, the average was 82.9 kg, with a minimum weight of 37 kg and a maximum of 180 kg.

**Table 1:** Prevalence of Nursing Diagnoses in COVID-19 survivors after hospitalization in a Critical Care Unit, Porto Alegre, Rio Grande do Sul, Brazil, 2022

Nursing Diagnoses	(n)	(%) total	(%) valid
*Impaired Physiological Balance Syndrome	1134	77.3	77.3
(00004) Risk for Infection	704	48	48
(00155) Risk for Falls	682	46.5	46.5
(00249) Risk for Pressure Injury	426	29	29
(00032) Ineffective Breathing Pattern	358	24.4	24.4
(00044) Impaired Tissue Integrity	323	22	22
(00030) Impaired Gas Exchange	320	21.8	21.8
(00033) Impaired Spontaneous Ventilation	290	19.8	19.8
(00206) Risk for Bleeding	218	14.9	14.9
*Self-Care Deficit Syndrome	217	14.8	14.8
(00132) Acute Pain	201	13.7	13.7
(00087) Risk for Perioperative Positioning Injury	154	10.5	10.5
(00108) Bathing Self-Care Deficit	106	7.2	7.2
(00245) Risk for Corneal Injury	98	6.7	6.7
(00046) Impaired Skin Integrity	95	6.5	6.5
(00047) Risk for Impaired Skin Integrity	94	6.4	6.4
(00179) Risk for Unstable Blood Glucose	80	5.5	5.5
(00002) Imbalanced Nutrition Less Than Body Requirements	73	5	5

*Nursing Diagnoses registered in the AGHUse system at Hospital de Clínicas de Porto Alegre due to a demand from nurses at the Critical Care Center, but not incorporated into the International Classification of Diagnoses (NANDA-I).

Table 1 shows the prevalence of ND in post-hospitalization COVID-19 survivors in a CCU. The prevalent diagnosis was Impaired Physiological Balance Syndrome (77.3%), followed respectively by (00004) Risk for Infection (48%), (00155) Risk for Falls (46.5%), (00249) Risk for Pressure Injury (29%), (00032) Ineffective Breathing Pattern (24.4%), (00044) Impaired Tissue Integrity (22%).

Of the diagnoses prescribed by nurses only once, the following stand out: (00105) Interrupted Breastfeeding; (00126) Deficient Knowledge; (00124) Hopelessness; (00256) Delivery Pain; (00052) Impaired Social Interaction; (00131) Impaired Memory; (00096) Sleep Deprivation; (00301) Maladaptive Grieving; (00139) Risk for Self-Mutilation; (00104) Ineffective Breastfeeding; (00290) Risk for Elopement Attempt; (00218) Risk for Adverse Reaction to Iodinated Contrast Media; and (00038) Risk for Physical Trauma.

It is noted that 9.9% of patients had changes in the level of consciousness such as confusion, lethargy, coma and agitation. Table 2 demonstrates the association of the level of consciousness/neurological regulation with the ND: (00128) Acute Confusion; (00129) Chronic Confusion; (00049) Decreased Intracranial Adaptive Capacity; (00201) Risk for Ineffective Cerebral Tissue Perfusion; (00103) Impaired Swallowing; (00123) Unilateral Neglect; (00131) Impaired Memory.

**Table 2:** Association between level of consciousness/neurological regulation with Nursing Diagnoses in COVID-19 survivors, Porto Alegre, Rio Grande do Sul, Brazil, 2022

Variable	Categories	Has at least (1) Nursing Diagnosis initiated		*p* value
		Yes	No	
Lucid				<0.001
	Yes	34 (4.0)	808 (96.0)	
	No	86 (13.8)	539 (86.2)	
Oriented				<0.001
	Yes	29 (3.9)	708 (96.1)	
	No	91 (12.5)	639 (87.5)	
Alert				0.083
	Yes	26 (6.1)	399 (93.9)	
	No	94 (9.0)	948 (91.0)	
Confused				<0.001
	Yes	24 (31.6)	52 (68.4)	
	No	96 (6.9)	1295 (93.1)	
Comatose				1.000
	Yes	2 (9.5)	19 (90.5)	
	No	118 (8.2)	1328 (91.8)	
Lethargic				<0.001
	Yes	10 (32.3)	21 (67.7)	
	No	110 (7.7)	1326 (92.3)	
Sedated				0.242
	Yes	15 (11.2)	119 (88.8)	
	No	105 (7.9)	1228 (92.1)	
Hectic				0.012
	Yes	5 (27.8)	13 (72.2)	
	No	115 (7.9)	1334 (92.1)	
Others*				0.007
	Yes	4 (40.0)	6 (60.0)	
	No	116 (8.0)	1341 (92.0)	

*Others refers to speech aphasia, anxiety, left hemiparesis, dysarthria, cerebral palsy, dyslalia.

**Figure 1:**
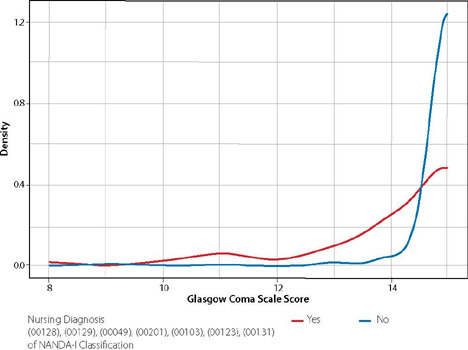
Association between Nursing Diagnoses and Glasgow score in post-Critical Care Unit COVID-19 survivors, Porto Alegre, Rio Grande do Sul, Brazil, 2022

Figure 1 shows the association between the Glasgow Scale score and the ND: (00128) Acute Confusion; (00129) Chronic Confusion; (00049) Decreased Intracranial Adaptive Capacity; (00201) Risk for Ineffective Cerebral Tissue Perfusion; (00103) Impaired Swallowing; (00123) Unilateral Neglect; and (00131) Impaired Memory. It should be noted that the average score of the Glasgow Scale was 14, with variations between 8 and 15.

**Figure 2:**
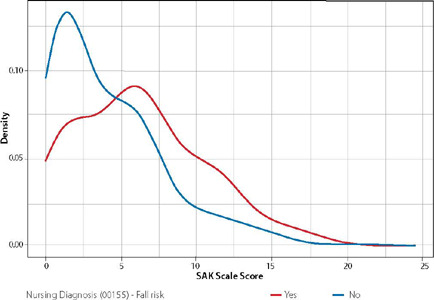
Association between the Nursing Diagnosis “Risk for Falls” and the SAK Scale score in post-Critical Care Unit COVID-19 survivors, Porto Alegre, Rio Grande do Sul, Brazil, 2022

The Mann-Whitney U test detects a difference between the Glasgow scores (p < 0.001), where those with at least one of the diagnoses described have a lower score. Figure 2 demonstrates the association between the SAK Scale score and the ND “Risk for Falls”. It should be noted that the mean SAK score in the analyzed sample was 5.3, with variations between 0 and 16.

The Mann-Whitney U test detects differences between SAK scores (p < 0.001), where those at risk for falling have a higher score. Table 3 shows the prevalence of nursing care in prescriptions for post-COVID-19 patients. A total of 564 nursing care procedures were identified, of which the following stand out: implementing aerosol precautions (94.5%); implementing droplet precautions (93.4%); checking vital signs (91.9%); applying standard disinfectant to equipment and surfaces (89.6%); implementing contact precautions (88.8%); implementing care according to the falls care protocol (82.9%).

**Table 3:** Prevalence of prescribed nursing care in COVID-19 survivors, Porto Alegre, Rio Grande do Sul, Brazil, 2022

Nursing care	(n)	(%)
Implementing aerosol precautions	1.387	94.5
Implementing droplet precautions	1.370	93.4
Checking vital signs	1.348	91.9
Applying standard disinfectant to equipment and surfaces	1.315	89.6
Implementing contact precautionary measures	1.302	88.8
Implementing care according to the fall care protocol	1.216	82.9
Keeping head elevated	1.196	81.5
Implementing venipuncture care	1.048	71.4
Communicating changes in the ventilatory pattern	1.001	68.2
Taking a bed bath	985	67.1
Implementing care protocol for pressure injury prevention and treatment	979	66.7
Checking oximetry	944	64.3
Performing oral hygiene by applying standard mouthwash	932	63.5
Implementing care with oxygen therapy (nasal cannula)	921	62.8
Applying 2% aqueous chlorhexidine to the body when changing cardiac electrode sets	810	55.2

Implementing aerosol and droplet precautions, with 94.5% and 93.5%, respectively, stand out. It should be noted that approximately 47% of care is related to the prevention of infections. Considering the above, the institutional concern is evident as well as that of the nursing team with Risk for Infection, which is the second most listed diagnosis in the analysis of the second stage of these patients’ NP.

## DISCUSSION

The severe form of COVID-19 demanded clinical reasoning and diagnostic judgment skills from nurses, aiming at the accurate application of NP to achieve results in the care provided to patients in critical care contexts^(4)^.

With regard to participants’ color/race, a higher prevalence of white people (81%) was identified, which corroborates the conclusions of a study developed in Ponta Grossa, Paraná (PR), in which more than 80% of patients diagnosed with COVID-19 were white^(14)^. This may be related to the predominance of whites in the population of Rio Grande do Sul (79%)^(15)^. This finding, therefore, cannot justify the lack of attention to the black population, whether in a pandemic context or not. This is because the difficulty of accessing health services still exists and needs to be overcome^(16)^. Thus, it is reaffirmed that society must include this population and seek results that guarantee equality, equity and comprehensive access to public health policies.

As for the results of the ND variable found in nurses’ records, the following stood out, respectively, as the most prevalent: Impaired Physiological Balance Syndrome (77.3%); (00004) Risk for Infection (48%); (00155) Risk for Falls (46.5%); (00249) Risk for Pressure Injury (29%); (00032) Ineffective Breathing Pattern (24.4%); and (00044) Impaired Tissue Integrity (22%). The ND, organized into domains and classes, can be defined according to the NANDA-I International Classification as “a clinical judgment concerning a human response to a health condition, vulnerability for that response, by an individual, family, group, or community”^(17)^. Thus, diagnoses can be focused on a problem, a risk, health promotion or even applied to syndromes.

In view of this, it can be seen that the prevalent diagnoses in the researched population, according to the above classification, are in Safety/Protection (Risk for Infection, Risk for Falls, Risk for Pressure Injury and Impaired Tissue Integrity) and Activity/Rest (Ineffective Breathing Pattern) domains. Regarding the classes, they are, respectively, infection, physical injury and cardiovascular/ pulmonary responses^(17)^.

Given the severity of the cases, it was evident that, in the sample under study, the focus of nursing problems during the pandemic was on psychobiological needs to the detriment of psychospiritual and psychosocial needs. A similar finding is evidenced in the study developed by the Nursing Process Research Network (RePPE - *Rede de Pesquisa em Processo de Enfermagem*) which identified the diagnoses Risk for Infection, Risk for Pressure Injury and Impaired Spontaneous Ventilation for hospitalized patients in critical condition^(4)^.

Among these diagnoses, it should be noted that the ND “Impaired Physiological Balance Syndrome”, which is in the process of being developed^(18)^, was identified in more than 70% of patients. It instigated, in this way, nurses to prescribe care in order to seek the physiological balance of the body of the individual being cared for. It should also be noted that the diagnosis mentioned above has not yet been incorporated into the NANDA-I Classification so far, but during the pandemic nurses working at the HCPA requested inclusion in the medical record system electronic AGHUse, seeking, therefore, to follow the other steps of NP according to this diagnosis. It should be noted that the scored diagnosis would reduce the time taken to carry out the process stages and would include numerous diagnoses present for severe cases of COVID-19^(18)^.

Additionally, it is pointed out that the diagnoses recorded by nurses only once, in the sample of this study, stand out: Interrupted Breastfeeding; Deficient Knowledge; Hopelessness; Delivery Pain; Impaired Social Interaction; Impaired Memory; Impaired Oral Mucous; Impaired Sleep Pattern; Grief; Risk for Aggression; Risk for Ineffective Breastfeeding; Risk for Elopement Attempt; Risk for Adverse Reaction to Iodinated Contrast Media; and Risk for Trauma.

With regard to neurological regulation/level of consciousness, it is known that COVID-19 caused transient and permanent neurological sequel, identified from clinical manifestations during and after hospitalization in CCU and observed by the health team. It is known that encephalitis, systemic inflammation, dysfunction of organs such as the liver, kidneys or lungs and cerebrovascular disorders can cause long-term neurological sequel, aggravating pre-existing symptoms or new cerebrovascular diseases^(3)^. Among the neurological manifestations, headache, anosmia, dysgeusia, dizziness, convulsions and impaired consciousness stand out^(3, 19)^.

According to the NANDA-I taxonomy, the ND frequently used for patients suffering from neurological conditions and who also have neurological consequences secondary to the disease were: Spiritual Distress; Hyperthermia; Acute Pain; Nausea; Unbalanced Nutrition Less Than Body Requirements; Impaired Swallowing; Acute Confusion; Risk for Acute Confusion; Impaired Memory; Disturbed Thought Process; Impaired Social Interaction; Fatigue; Anxiety; Impaired Urinary Elimination; Constipation; Risk for Constipation; Diarrhea; Impaired Physical Mobility; Feeding Self-Care Deficit; Bathing Self-Care Deficit; Toileting Self-Care Deficit; Dressing Self-Care Deficit; Risk for Relocation Stress Syndrome; Risk for Infection; Risk for Falls; Risk for Pressure Ulcer; Risk for Impaired Skin Integrity; Impaired Skin Integrity; Ineffective Protection; Ineffective Breathing Pattern; Impaired Gas Exchange; Impaired Spontaneous Ventilation; Excessive Fluid Volume; and Risk for Electrolyte Imbalance^(17, 18, 20)^.

Based on this premise, we sought to verify the association between the Glasgow Scale score and the ND related to the NANDA-I domains, focusing on the level of consciousness and neurological regulation: (00128) Acute Confusion; (00129) Chronic Confusion; (00049) Decreased Intracranial Adaptive Capacity; (00201) Risk for Ineffective Cerebral Tissue Perfusion; (00103) Impaired Swallowing; (00123) Unilateral Neglect; and (00131) Impaired Memory.

The Glasgow Coma Scale (GCS), developed in 1974 at the University of Glasgow, Scotland, by Graham Taeasdaale and Bryan Jennet, is used around the world to identify neurological disorders and monitor the evolution of level of consciousness, in addition to predicting prognosis and standardize health professionals’ language regarding the assessment of patients’ level of consciousness. The scale’s total score can vary between 3 and 15 points, being obtained from the assessment of spontaneous activities and the application of verbal and/or painful stimuli. The scale is composed of three domains that assess patients’ eye opening, verbal response and motor response^(21)^.

It should be noted that the mean score on the GCS was 14, with variations between 8 and 15. The mean score on the GCS demonstrates that confusion was present in most patients, which may be related to delirium after prolonged sedation.

It is noted that 9.9% of patients had changes in the level of consciousness such as confusion, lethargy, coma and agitation. There was a significant association between the 842 patients with the “lucid” status and 34 (4.0%) had at least one of the 7 diagnoses related to the level of consciousness and neurological regulation, while those who did not have the “lucid” status (13, 8%) had at least one of the 7 diagnoses. The independence chisquare test detected an association between the “lucid” status and having at least one of the diagnoses (p < 0.001). Thus, 120 (8.2%) of patients in the study had at least one of the 7 diagnoses. Such evidence denotes that patients who had lucidity in their level of consciousness presented risk or some type of alteration in the level of consciousness during or after hospitalization in the CCU, corroborating with the studies developed in São Paulo and Pernambuco that identified post-COVID-19 neurological changes^(3,19)^.

Regarding the SAK Scale score and the ND “Risk for Falls”, it is noteworthy that the mean SAK score in the analyzed sample was 5.3 with variations between 0 and 16. The SAK Fall Scale, developed by Severo, Almeida and Kuchenbecker includes seven variables: disorientation/confusion, frequent urination, walking limitations, lack of caregiver, postoperative status, previous falls and number of medications administered within 24 hours prior to the fall. Scores using the scale’s scoring system classify patients into risk classes such as: low risk: less than or equal to 6.0; moderate risk: 6.5 to 10.0; and high risk: greater than or equal to 10.5^(22)^. The association between the SAK score and the ND “Risk for Falls” was significant, demonstrating that nurses identified the diagnosis for all patients with moderate or high scores after assessing risk for falls using the SAK scale.

Of the fifteen prevalent nursing care identified in the nursing prescriptions, it is noteworthy that approximately 47% of care is related to infection prevention. Given the above, the institutional concern as well as that of the nursing team with Risk for Infection is evident, with this diagnosis being the second most listed in the analysis of the second stage of the NP implemented for patients, following the RePPE guidelines for patients with COVID-19 admitted to a CCU^(4)^.

### Study limitations

With regard to the study limitations, it should be noted that the results of this study cannot be generalized, as they correspond to those found in patients’ medical records. To this end, multicenter studies would be needed. Still, the scarcity of studies aimed at applying the ND process in pandemic situations was identified. Thus, new qualitative research is needed in order to identify nurses’ perceptions in relation to the low selection of ND related to principle of life domains and value class, beliefs and coherence between values, beliefs and acts.

### Contributions to nursing and health

The analysis of sociodemographic profile, diagnoses and the most prevalent nursing care identified in this study allows nursing to know the focus that has been given to patient care. Furthermore, it provides notes for the need to identify/investigate psychosocial and psychospiritual needs in implementing NP and its consequent registration in pandemic contexts such as the coronavirus.

## CONCLUSIONS

COVID-19 affected mostly white men, married and retired, aged 58 years. Diagnoses and nursing care have shown to contribute to quality of care in clinical nursing practice, with emphasis on ND: Impaired Physiological Balance Syndrome (77.3%); Risk for Infection (48%); Risk for Falls (46.5%); Risk for Pressure Injury (29%); and Ineffective Ventilatory Pattern (24.4%). The overvaluation of psychobiological needs dimensions was evidenced to the detriment of psychospiritual and psychosocial needs, which is a gap to be considered by the nursing team in the context of clinical practice, in particular, in the face of pandemics, as it is a context of care in which these are revealed.

In this regard, it is important to teach clinical reasoning and holistic critical thinking skills for future nurses, preparing them for a global nursing assessment to meet all dimensions of patients’ basic human needs, in the light of a theory, above all, of nursing. The study findings may support clinical practice and the application of the diagnostic process by nurses in pandemic situations, such as the coronavirus. The evidence found guides the NP stages accurately, which denotes the application of critical thinking and clinical reasoning skills in nurses’ clinical judgment in care practice. Patients who are admitted to the CCU require specific care upon discharge to the inpatient unit. Considering the coronavirus infection, care should be redoubled and focused on complications arising from the disease.

The ND process becomes an ally of nurses, enabling a critical and holistic look at patients’ needs in pandemic contexts. The research showed the need for continuous education for the team for a human and holistic view without neglecting psycho-spiritual and psychosocial needs, consequently the diagnoses focused on the domain principles of life of the International Classification of Diagnostics (NANDA-I) associated with diagnoses with a focus on in psychobiological needs.
